# Supplementary material for: Rough and smooth variants of Mycobacterium abscessus are differentially controlled by host immunity during chronic infection of adult zebrafish
Source: Nat Commun. 2022 Feb 17;13:952. doi: 10.1038/s41467-022-28638-5 (PMC8854618; doi:10.1038/s41467-022-28638-5)
Supplement: Supplementary file 1 — Supplementary Information [file 41467_2022_28638_MOESM1_ESM.pdf]

Supplementary Information

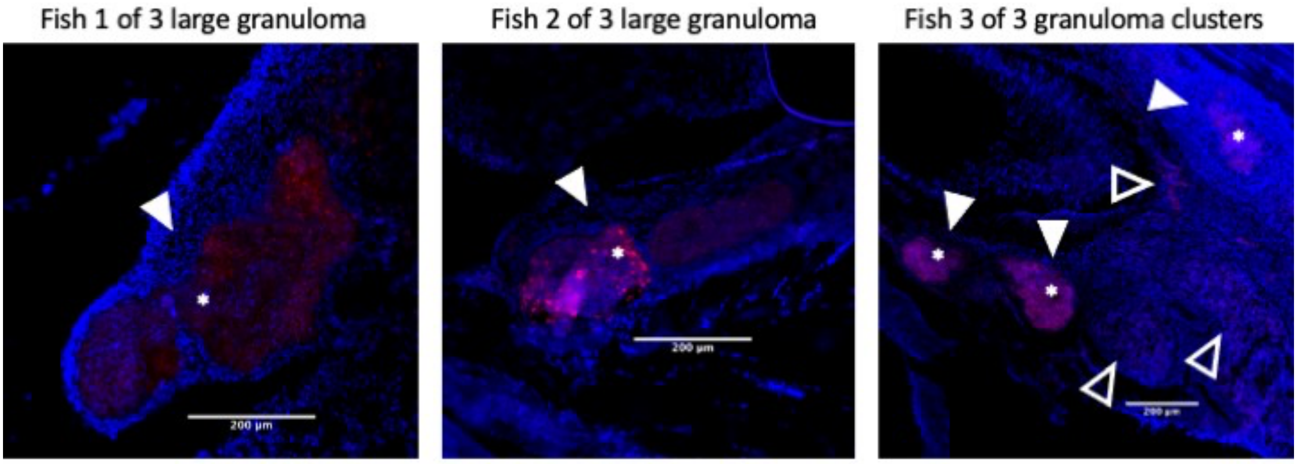

Supplemental Figure 1: Persistence of fluorescent *M. abscessus* at 10 weeks post infection
Representative images of R *M. abscessus*-tdTomato lesions in DAPI-stained cryosections from three 70 dpi adult zebrafish. Filled arrowheads indicate necrotic granulomas, * indicate necrotic cores, empty arrowheads indicate cellular *M. abscessus* granulomas. Scale bars indicate 200 µm.

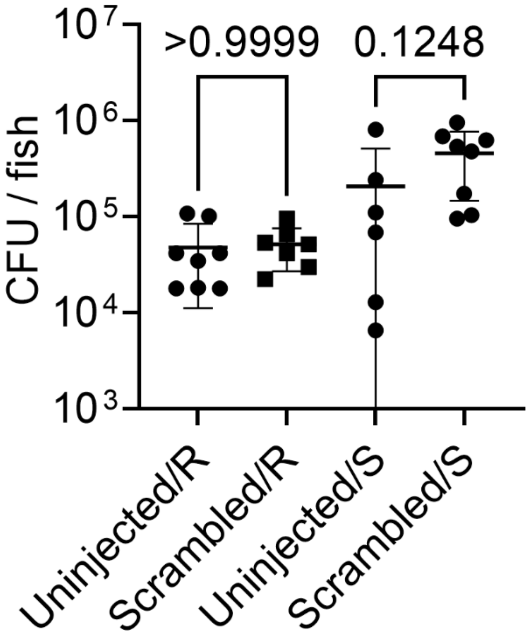

Supplemental Figure 2: Injection of scrambled gRNA/Cas9 does affect *M. abscessus* bacterial burden. Enumeration of CFUs from uninjected and scrambled gRNA/Cas9-injected adult zebrafish infected with *M. abscessus* R or *M. abscessus* S. Each data point represents a single animal from a single experiment, total number of animals per column = 8/7/6/8. Statistical testing by two-sided ANOVA. Data are presented as mean values +/- SD. Source data are provided in the Source Data file.

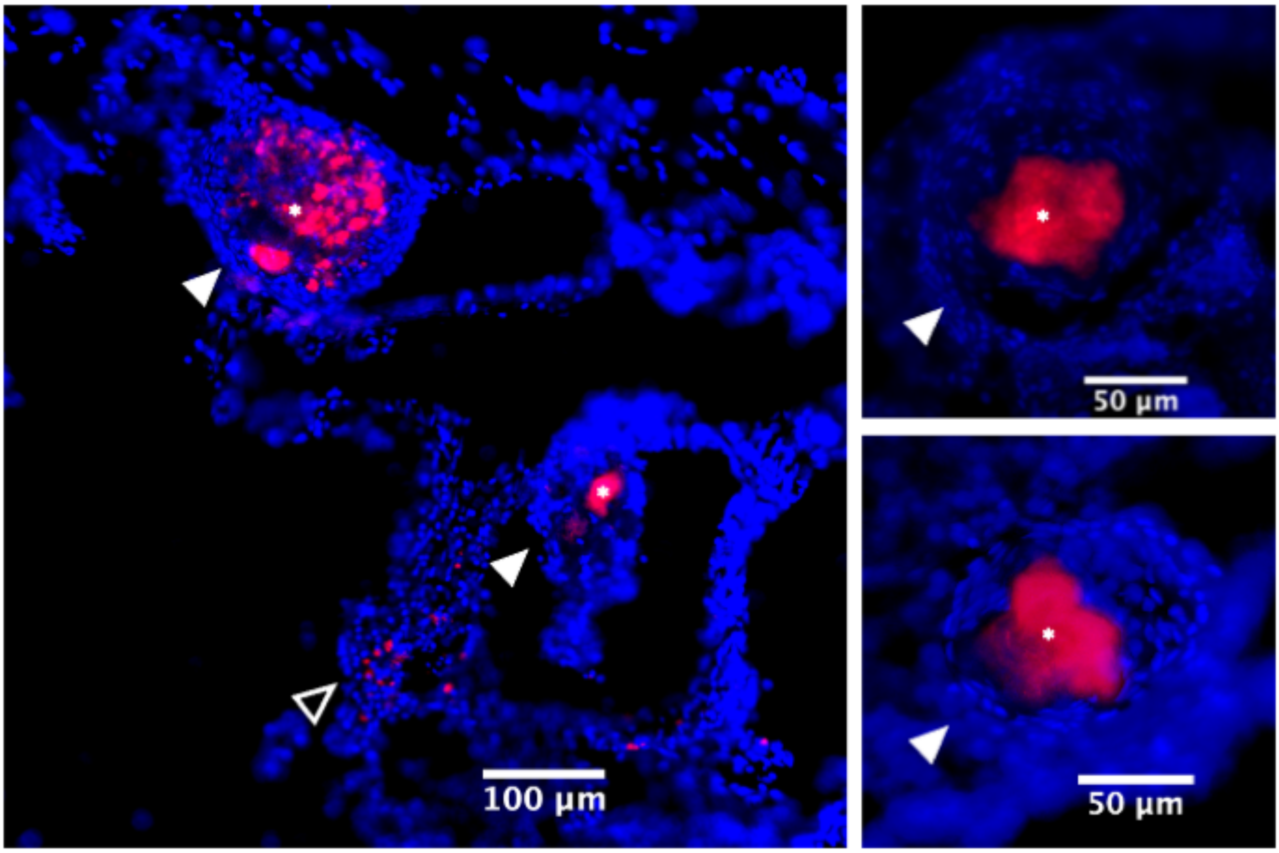

Supplemental Figure 3: Formation of small granulomas in T cell-deficient adult zebrafish. Representative images of R *M. abscessus*-tdTomato lesions in DAPI-stained cryosections from a 56 dpi *lck*$^{-/-sa410}$ fish. Filled arrowheads indicate necrotic granulomas, * indicate necrotic cores, empty arrowheads indicate cellular *M. abscessus* granulomas.
