# Supplementary material for: Rough and smooth variants of Mycobacterium abscessus are differentially controlled by host immunity during chronic infection of adult zebrafish
Source: Nat Commun. 2022 Feb 17;13:952. doi: 10.1038/s41467-022-28638-5 (PMC8854618; doi:10.1038/s41467-022-28638-5)
Supplement: Supplementary file 3 — Reporting Summary [file 41467_2022_28638_MOESM3_ESM.pdf]

nature research

Corresponding author(s): Stefan Oehlers

Last updated by author(s): Jan 19, 2022

Reporting Summary

Nature Research wishes to improve the reproducibility of the work that we publish. This form provides structure for consistency and transparency in reporting. For further information on Nature Research policies, see our Editorial Policies and the Editorial Policy Checklist.

Statistics

For all statistical analyses, confirm that the following items are present in the figure legend, table legend, main text, or Methods section.

n/a	Confirmed	
☐	☒	The exact sample size (*n*) for each experimental group/condition, given as a discrete number and unit of measurement
☐	☒	A statement on whether measurements were taken from distinct samples or whether the same sample was measured repeatedly
☐	☒	The statistical test(s) used AND whether they are one- or two-sided<br>*Only common tests should be described solely by name; describe more complex techniques in the Methods section.*
☒	☐	A description of all covariates tested
☐	☒	A description of any assumptions or corrections, such as tests of normality and adjustment for multiple comparisons
☐	☒	A full description of the statistical parameters including central tendency (e.g. means) or other basic estimates (e.g. regression coefficient) AND variation (e.g. standard deviation) or associated estimates of uncertainty (e.g. confidence intervals)
☐	☒	For null hypothesis testing, the test statistic (e.g. *F*, *t*, *r*) with confidence intervals, effect sizes, degrees of freedom and *P* value noted<br>*Give P values as exact values whenever suitable.*
☒	☐	For Bayesian analysis, information on the choice of priors and Markov chain Monte Carlo settings
☒	☐	For hierarchical and complex designs, identification of the appropriate level for tests and full reporting of outcomes
☒	☐	Estimates of effect sizes (e.g. Cohen's *d*, Pearson's *r*), indicating how they were calculated

Our web collection on statistics for biologists contains articles on many of the points above.

Software and code

Policy information about availability of computer code

Data collection: Images were collected using LAS software to operate a Leica DM6000B microscope. CFX Maestro software to operate the Biorad CFX qPCR machine.

Data analysis: Images were analysed in ImageJ. Statistical comparisons and graphs were prepared in Graphpad Prism.

For manuscripts utilizing custom algorithms or software that are central to the research but not yet described in published literature, software must be made available to editors and reviewers. We strongly encourage code deposition in a community repository (e.g. GitHub). See the Nature Research guidelines for submitting code & software for further information.

Data

Policy information about availability of data

All manuscripts must include a data availability statement. This statement should provide the following information, where applicable:

- Accession codes, unique identifiers, or web links for publicly available datasets
- A list of figures that have associated raw data
- A description of any restrictions on data availability

The processed analysis data are included in the Source Data file that is provided with this paper. Raw image files will be archived for 10 years (2022-2032) by The Centenary Institute (Sydney, Australia). The raw image and analysis data are available under restricted access due to their large size and prohibitive cost of public hosting: they can be obtained upon request by email from the corresponding author, who will mediate access from the Centenary Institute servers. Expected response times will be within one week.

The raw images acquired for this manuscript are in the hundreds of gigabytes to terabyte range of size. It is technically possible to make subsets of these files available on demand via online file sharing but it would be prohibitively expensive to post the entire cache of images on a public server.

Field-specific reporting

Please select the one below that is the best fit for your research. If you are not sure, read the appropriate sections before making your selection.

☒ Life sciences ☐ Behavioural & social sciences ☐ Ecological, evolutionary & environmental sciences

For a reference copy of the document with all sections, see nature.com/documents/nr-reporting-summary-flat.pdf

Life sciences study design

All studies must disclose on these points even when the disclosure is negative.

Sample size

Sample sizes were estimated from historical data with Mycobacterium marinum infection of adult zebrafish to detect half log differences in bacterial burden between two groups with a standard deviation of half the larger number. An online power calculator yielded group sizes of at least 7 for 0.8 beta and 0.05 alpha parameters.

Data exclusions

Data were excluded by ROUT outlier analysis with a 1% stringency threshold for analyses of individual granulomas. This outlier analysis had the effect of removing regions of interest that had unusually high or low bacterial or host cell fluorescence. The exclusion did not affect the statistical test results but provide a narrower range of data points in the graphs (Figures 3C, 5C, 6C).

Replication

Experiments were replicated as indicated throughout the manuscript.
Figure 1: 3 experimental replicates, Figure 2: 2 experimental replicates, Figure 3C: 1 experiment with individually analysed animals 3/2/3/3 per column, Figure 3D: 2 experimental replicates, Figure 4A: 2 experimental replicates, Figure 4B: 3 experimental replicates, Figure 4C and 4D: 2 experimental replicates, Figure 4F: 3 experimental replicates, Figure 4G and 4H: 2 experimental replicates, Figure 5C: 1 experiment with individually analysed animals 2/3/3/2 per column, Figure 5D: 2 experimental replicates, Figure 5E: 3 experimental replicates, Figure 6C: 1 experiment with individually analysed animals 4/3/3/3 per column, Figure 6D: 2 experimental replicates, Figure 6E and 6F: 2 experimental replicates.

Randomization

Organisms were randomly assigned into infection or drug treatment groups by netting of live fish.

Blinding

Blinding was not carried out at the data collection or analysis stages. The data collection and analyses were performed by different researchers for Figures 4 and 6, otherwise collection and analysis was carried out by a single researcher. Resources were not available for the hiring of sufficient staff to separate collection and analysis across the length of the project.

Reporting for specific materials, systems and methods

We require information from authors about some types of materials, experimental systems and methods used in many studies. Here, indicate whether each material, system or method listed is relevant to your study. If you are not sure if a list item applies to your research, read the appropriate section before selecting a response.

Materials & experimental systems

n/a	Involved in the study
☐	☒ Antibodies
☒	☐ Eukaryotic cell lines
☒	☐ Palaeontology and archaeology
☐	☒ Animals and other organisms
☒	☐ Human research participants
☒	☐ Clinical data
☒	☐ Dual use research of concern

Methods

n/a	Involved in the study
☒	☐ ChIP-seq
☒	☐ Flow cytometry
☒	☐ MRI-based neuroimaging

Antibodies

Antibodies used

Chicken anti-GFP (ab13970, Abcam), Goat anti-chicken AF488 conjugate (ab150173, Abcam)

Validation

https://www.abcam.com/gfp-antibody-ab13970.html - control and GFP overexpressing cell validation
https://www.abcam.com/goat-chicken-igy-hl-alexa-fluor-488-preadsorbed-ab150173.html - By immunoelectrophoresis and ELISA this antibody reacts specifically with chicken IgY and with light chains common to other chicken immunoglobulins. No antibody was detected against non-immunoglobulin serum proteins. Reduced cross-reactivity to bovine, goat, horse, human, mouse, pig, rabbit and rat IgG was detected. This antibody may cross react with IgG from other species.

Animals and other organisms

Policy information about studies involving animals; ARRIVE guidelines recommended for reporting animal research

Laboratory animals	Danio rerio, AB strain, mixed male and female sexes were used, animals were between 2 and 12 months old. Strains: AB strain wildtype, TgBAC(tnfa:GFP)pd1028, TgBAC(lck:EGFP)vcc4, lck-/- sa410 mutant, TgBAC(foxp3a:TagRFP,cryaa:EGFP)vcc3, foxp3avcc6 mutant
Wild animals	No wild animals were used in the study.
Field-collected samples	No field collected samples were used in the study.
Ethics oversight	Sydney Local Health District Animal Welfare Committee

Note that full information on the approval of the study protocol must also be provided in the manuscript.
